# Spread of *Chlamydia pneumoniae* ST16 during the 2024–2025 outbreak in France

**DOI:** 10.1080/22221751.2026.2703399

**Published:** 2026-07-11

**Authors:** Arabella Touati, Cécile Bébéar, Olivia Peuchant, the CHLAPTY study group

**Affiliations:** a Department of Bacteriology, National Reference Centre for bacterial Sexually Transmitted Infections, Bordeaux, France; b UMR 5234 CNRS Microbiologie Fondamentale et Pathogénicité (MFP), ARMYNE, Univ. Bordeaux, Bordeaux, France

**Keywords:** *Chlamydia pneumoniae*, multi-locus sequence typing, respiratory infection, outbreak, genotypic distribution

## Abstract

A resurgence of *Chlamydia pneumoniae* respiratory infections was observed in France during 2024–2025. We aimed to characterize the genotypic distribution of *C. pneumoniae* during the outbreak period using positive respiratory samples from infected individuals. Molecular typing was performed by applying the Chlamydiales Multi-Locus Sequence Typing (MLST) scheme. Our results revealed the exclusive predominance of *C. pneumoniae* ST16, suggesting dissemination of ST16 across France. Further molecular analyses of isolates from other countries are needed to determine whether the recent resurgence of *C. pneumoniae* infections is driven by clonal expansion.

*Chlamydia pneumoniae* is a known cause of community-acquired pneumonia (CAP). The clinical course of infection varies from subclinical to mild illness and, more rarely, to severe pneumonia [1]. *C. pneumoniae* infection has also been associated with the initiation and exacerbation of asthma and has been implicated in the pathogenesis of atherosclerosis [2]. Respiratory infections caused by *C. pneumoniae* affect all age groups but are more common in children. Before the COVID-19 pandemic, sporadic outbreaks of *C. pneumoniae* have been documented in closed communities, such as in 2014 in a prison in Texas and in 2016 in children in South Korea [3,4]. The post-COVID period has been marked by a resurgence of several respiratory pathogens, such as *Mycoplasma pneumoniae, Bordetella pertussis* and *Streptococcus pyogenes* [5–7]. Molecular studies of these pathogens showed that these upsurges were not associated with the emergence of a single clone but, rather, with the circulation or predominance of several types [5–7]. With a slight delay, a rebound in *C. pneumoniae* detection was observed in European and Asian countries, by the end of 2023 [8–10]. During routine epidemiologic surveillance at Bordeaux University Hospital, France, *C. pneumoniae* PCR positivity reached 1.94% in 2024, peaking at 4.5% November 2024, compared with 0-0.66% over the preceding decade. In 2024, the French National Network of Hospital Laboratories (RENAL network) similarly reported a rise in *C. pneumoniae* PCR detection between July and September 2024, with positivity increasing from 1.0% to 2.3%, and reaching 3.1% in November, which is more than threefold higher than during the same period in the previous year. To date, no data are available to determine whether the resurgence of *C. pneumoniae* respiratory infections resulted from the spread of a single or multiple strain(s). *C. pneumoniae* genotyping can be performed by sequencing the variable domain IV (VD4) of the *omp*A gene, for which four genotypes (A to D) have been described [2]. Another method is Chlamydiales multilocus sequence typing (MLST), a scheme developed to investigate the genetic diversity of these species [11]. The aim of this study was to characterize the genotypic distribution of *C. pneumoniae* during the outbreak period in France by analysing positive respiratory samples from infected individuals.

We retrospectively collected respiratory specimens that tested positive for *C. pneumoniae* from January 2024 to May 2025 from nine regions across mainland France (Fig S1). Epidemiological and clinical data were extracted from medical records. *omp*A genotyping for *C. pneumoniae* was performed by sequencing a 366-bp fragment of the VD4 region of this gene [2]. The Chlamydiales MLST scheme was also used to discriminate isolates further [11]. This approach targets seven housekeeping genes (*gat*A, *opp*A_3, *hfl*X, *gid*A, *eno*A, *hem*N, and *fum*C). Target genes were amplified and sequenced using primers and conditions described on the Chlamydiales MLST website (https://pubmlst.org/organisms/chlamydiales-spp). Numbers for alleles and sequence types (STs) were assigned in accordance with the Chlamydiales MLST Database.

Overall, we collected and analysed 123 respiratory specimens positive for *C. pneumoniae*. The number of cases peaked in September and October 2024 (Figure 1A). The female-to-male ratio was 1.08. The median age was 13 years [6.5–34.5]. Children aged ≤ 15 years represented 54.5% (67/123) of cases, while 22% (27/123) occurred in individuals aged 16–35 years and 23% (29/123) in those aged 36–80 years (Figure 1B). Among the 93 individuals with available clinical data, nearly one third (39.8%, 37/93) had community-acquired pneumonia and the remaining reported symptoms of rhinitis, prolonged cough, wheezing or asthma exacerbation. Fever was present in most cases (63.8%, 44/69). *omp*A genotyping for *C. pneumoniae* was successful for 85 samples and all sequences obtained identical and corresponded to genotype A (Genbank accession number AE002161), the most common genotype associated with human infections worldwide [2]. The Chlamydiales MLST scheme was then applied to all specimens including those with an undetermined *omp*A genotype [11]. Among them, none of the seven genes were amplified in 20, and 47 samples presented an incomplete ST profile with only one to six loci amplified and alleles belonging to ST16. A complete sequence type (ST) was obtained for 56 specimens, all of which were identified as ST16. These isolates also belonged to *omp*A genotype A. The demographic (sex ratio, age distribution), monthly distribution over the study period, and geographic characteristics of these 56 *C. pneumoniae*–positive specimens were comparable to those of the overall cohort (Figure 1, Fig S1). The clinical characteristics of cases with undetermined ST and those that were successfully typed were similar, including disease duration, sampling site and prior antibiotic exposure. By contrast, the cycle threshold (Ct) values for the *C. pneumoniae* PCR were significantly higher in samples for which typing was undetermined than in the ST16 samples (median, 37.9 [34.8–40.0] vs 30.5 [27.7–34.5], respectively; *p* < 0.001). These findings suggest that a low bacterial load impaired successful typing.

**Figure 1.**
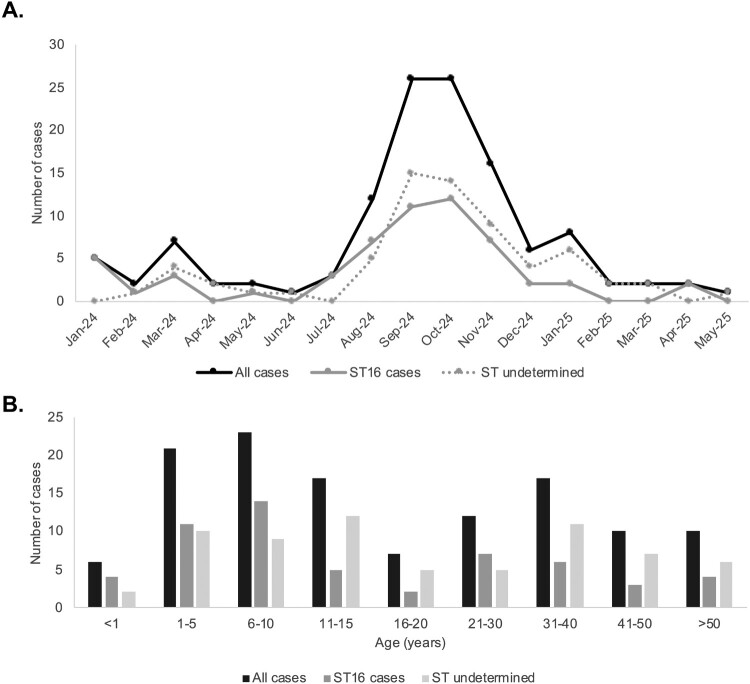
Distribution of all *C. pneumoniae* cases, ST16 cases and undetermined ST cases by month (A) and by age (B).

Our study highlights the spread of *C. pneumoniae* ST16 during the 2024–2025 outbreak in France. Studies about *C. pneumoniae* molecular typing are scarce. Based on currently available *C. pneumoniae* sequence data, MLST distinguishes three STs associated with human infections: ST16, ST17, and ST18. Among the *C. pneumoniae* genome sequences available in public databases, a complete ST profile could be assigned to 21 genomes. ST17 was the most frequently reported and has been identified in respiratory (*n* = 9), cardiovascular (*n* = 5), and conjunctival (*n* = 1) specimens. Only five isolates from respiratory samples have been assigned the ST16. ST 18 has been reported only once, in an isolate obtained from respiratory samples in Finland. In the present study, all successfully typed isolates belonged to ST16, indicating that this ST predominated during the study period. This finding is consistent with previous analyses of four full-length *C. pneumoniae* genomes obtained from positive respiratory samples in Marseille, France, which were ST16 and phylogenetically closely related to each other [8]. ST16 was also recently identified in respiratory samples from four individuals with severe pneumonia in Lishui City, Zhejiang Province, China collected between April and May 2024 [12].

During the COVID-19 pandemic, non-pharmaceutical interventions, such as lockdowns, social distancing, masks-wearing and school disclosure, markedly reduced the circulation of *C. pneumoniae*. Consequently, cohorts of children and adults experienced fewer natural exposures and fewer immunological boosting events. This resulted in a population-level immunity gap or accumulation of susceptible individuals, corresponding to an immunity debt. When social mixing resumed, many populations exhibited an unusually large pool of susceptible individuals, facilitating outbreaks of *C. pneumoniae* [8–10]. Additional factors probably amplified the apparent resurgence of *C. pneumoniae*. The often mild or non-specific clinical presentation of *C. pneumoniae* infections allows for prolonged undetected community transmission before outbreaks become clinically apparent. Furthermore, the predominance of *C. pneumoniae* ST16 cases might reflect emergence of a fitter variant.

Our study has several limitations. First, the analysis was restricted to cases that occurred between January 2024 and May 2025. It would have been informative to analyse *C. pneumoniae*-positive samples collected prior to 2024, in order to determine whether a different ST had been circulating previously. Second, 47 samples had an incomplete ST profile, which may be related to a low bacterial load in the specimens. Alternative methods such as amplicon enrichment approaches coupled with next-generation sequencing might allow for better typing efficiency and resolution.

To our knowledge, this study represents the largest molecular typing study of *C. pneumoniae* conducted on respiratory samples to date. Whole genome sequencing of *C. pneumoniae*-positive samples from this study and from other countries is required in order to conduct phylogenetic dating and test hypotheses of clonal expansion.

## Supplementary Material

Figure S1 revised mansucript.docx
