# Supplementary material for: Spread of Chlamydia pneumoniae ST16 during the 2024–2025 outbreak in France
Source: Emerg Microbes Infect. 2026 Jul 11;15(1):2703399. doi: 10.1080/22221751.2026.2703399 (PMC13417648; doi:10.1080/22221751.2026.2703399)
Supplement: Figure S1 revised mansucript.docx [file TEMI_A_2703399_SM6113.docx]

**Nouvelle-Aquitaine**

**Provence-Alpes**

**Côte-d’Azur**

**Occitanie**

**Auvergne-Rhône-Alpes**

**Bourgogne-Franche-Comté**

**Hauts-de-France**

**Bretagne**

**Pays de la Loire**

**Ile-de-France**

**Figure S1.** Geographical distribution of *C. pneumoniae* and ST16 cases in mainland France.

The undetermined *C. pneumoniae* ST cases are not represented.

1

2-5

9-12

**Number of ST16 cases**

1-10

11-20

21-30

**Number of *C. pneumoniae* cases**

1

2-5

9-12
